# Mixed-Method Follow-Up of Toddler-Aged Children with Spastic Cerebral Palsy After an Intense Physical/Occupational Therapy Intervention

**DOI:** 10.3390/children13030321

**Published:** 2026-02-25

**Authors:** Heidi L. Pottinger, Nicole P. Yuan, Burris Duncan

**Affiliations:** 1 Department of Health Promotion Sciences, Mel and Enid Zuckerman College of Public Health, University of Arizona, Tucson, AZ 85724, USA; 2 Department of Pediatrics, College of Medicine, University of Arizona, Tucson, AZ 85724, USA; 3 Sonoran Center for Excellence in Disabilities, University of Arizona, Tucson, AZ 85724, USA

**Keywords:** cerebral palsy, Perception-Action Approach, mixed methods, evaluation, parents’ perceptions

## Abstract

**Highlights:**

**What are the main findings?**

- Following an intense physical and occupational therapeutic intervention, based on prognostic GMFM-66 developmental curves by age for children with cerebral palsy, seven of 17 toddler-age children showed greater than expected improvement (all 5 with GMFCS II), four met the expected improvement, and six did not.
- Caregivers reported that the intense intervention contributed to the improvements in their children’s functioning.

**What is the implication of the main findings?**

- Prognostic predictions of functional outcomes in children with cerebral palsy are problematic.
- More rigorous mixed methods research is needed to evaluate the long-term impacts of intense, early interventions for children with spastic cerebral palsy.

**Abstract:**

Background/Objectives: A plethora of articles report the effectiveness of many different interventions for managing cerebral palsy (CP), but there are few long-term follow-up studies of children after an intervention designed to improve function in children with CP. This observational mixed-methods study examined the functional gains observed more than one year after toddlers completed a 48-week investigation that included 5 days per week for 12 weeks of occupational and physical therapy using the Perception-Action Approach (P-AA). The aim was to observe whether the functional gains made by the children continued to improve, plateaued, or declined at long-term follow-up. Methods: The sample was 23 children with a mild-to-moderate level of CP (Gross Motor Function Classification System I, II, or III) who completed the original study at least one year prior. The follow-up assessment included quantitative data using the Gross Motor Function Measure-66 (GMFM) and the Pediatric Evaluation of Disability Inventory-Functional Skills (PEDI-FS). Seventeen of 23 children were evaluated with both instruments. Qualitative data were collected from 14 of the 23 families who completed the PEDI-FS. Those families completed a survey with an open-ended questionnaire that assessed the caregivers’ perspectives about their children’s functioning and the impact of the intervention. Results: Findings from the quantitative data based on prognostic GMFM-66 developmental curves by age for children with CP: seven of the 17 children who had GMFM evaluations showed greater than expected improvement (all 5 with GMFCS II), four met the expected improvement, and six did not. Children with GMFCS II or III maintained their positions relative to the mean on the PEDI-FS mobility subset. Findings from the qualitative data revealed that some parents believed the intervention contributed to the changes in their children’s physical, mental, and social functioning. Many parents indicated that the study helped them overcome financial barriers related to accessing intensive therapies. Most parents reported that their child’s functioning was better than they expected when given the diagnosis of CP. Conclusions: Many months following an intense physical and occupational therapeutic intervention, based on predicted age-appropriate percentiles for motor function, roughly one-third of the children exceeded expectations, and one-third did not meet expectations. Despite the time invested in the intense protocol, caregivers felt the intervention was largely responsible for improvements in their children’s functioning.

## 1. Introduction

Cerebral Palsy (CP) describes a group of disorders in children that affect the development of movement, balance, and posture, causing limited activity. These disorders are attributed to non-progressive injuries or insults that occur in the developing fetal or infant brain [1]. Cerebral palsy is the most frequent childhood motor disability, with a prevalence in the United States ranging from 3.1 to 3.6 per 1000 live births [2]. The insult can occur during pregnancy, at the time of birth, or shortly after birth. The type and location of the motor dysfunction are dependent on the area of the brain that has been injured. The type can be hypertonic or spastic (the most frequent type), dyskinetic, marked by slow or rapid uncontrolled movements, ataxic, where balance is disturbed, or a mixture of these three types. The disability can be mild or severe and correlates with the size, location of the brain injury, and the neuronal developmental events occurring at the time of the central nervous system insult. CP can involve one, two, three, or all four extremities, or one side of the body (hemiplegia). The severity level is based on the ability to ambulate, determined by the Gross Motor Function Classification System (GMFCS) [3]. The severity varies from Level I, where a child can walk independently without the need for any external devices, to Level V, where the child needs a wheelchair and is unable to ambulate independently.

In 2019, Novak et al. published a systematic review of systematically reviewed articles providing an overview of the evidence of the effectiveness of a large variety of different interventions for children with CP. They identified 1584 citations, of which 247 met their criteria for inclusion. Some interventions consisted of strategies designed to prevent CP, while others focused on the management of CP. They categorized the interventions according to the degree of effectiveness. Common elements of effective interventions to improve motor function included: high-intensity, real-life tasks with goals set by the child or parent proxy, home programs, child motivation, and environmental enrichment to promote task performance; principles of neuroplasticity [4].

Our team completed a 48-week, randomized, parallel group trial design clinical trial examining the effectiveness of an intense regimen of the Perception-Action Approach [5,6] which employs most of the important elements of effective interventions identified by Novak et al. [4]. The sample consisted of 90 children with spastic CP, between 12 and 36 months of age (at time of consent), with a mild to moderate severity level as categorized by the Gross Motor Function Classification System. Randomization was stratified by age at the first therapeutic intervention (12–24 or 25–38 months) and severity level (GMFCS I, II, or III) and balanced by study site [5]. P-AA was not evaluated or included by Novak et al.’s systematic review of systematic reviews of CP interventions because there are no published systematic reviews of the P-AA. P-AA incorporates many elements that enhance neuroplastic changes: active, not passive therapy, repetition, child-directed (self-generated) active movements administered with high-intensity. In P-AA, the therapist adapts to the child’s schedule, interests, and initial movements and lightly assists the child’s movement to form new movement solutions; it is active, not passive, therapy [6].

Although there is a plethora of investigations designed to improve function in children with CP, few publications enrolled toddler-aged children, and there are few follow-up studies. This current report is an observational follow-up, mixed-method analysis of a convenience sample of 23 children who enrolled in the original study [5]. Each child had completed the 48-week protocol, had both a baseline and a 48-week evaluation, and a follow-up assessment at least one year after the 48-week evaluation.

The primary objective of this observational report was to evaluate the functionality of children with CP sometime after they had completed an intervention designed to improve function.

This study focused on the following five research questions. The first two questions were addressed by quantitative data, and the last three questions by qualitative data. No hypotheses are provided because this was a descriptive study.

1. Did the functional gains seen at the end of the original study continue to improve, plateau, or decline sometime after the 48 weeks, based on quantitative changes in the GMFM and/or the PEDI-FS assessed for each of the three GMFCS severity levels (I, II, or III)?
2. How did the changes compare to published developmental curves for both evaluation assessments?
3. What were the parents’ self-reported perceptions of the physical, mental, and social functioning of their child after completing the original study’s 48-week protocol?
4. What were the parents’ perceptions of barriers to treatment?
5. What were the parents’ expectations at the time they were given the diagnosis of cerebral palsy of their children’s later functioning?

## 2. Methods

### 2.1. Participants

This was an observational study, involving a follow-up sample of 23 children who completed the 48-week protocol of the original study from three different sites in Arizona: thirteen from Tucson Medical Center (TMC) in Tucson, nine from Phoenix Children’s Hospital (PCH) in Phoenix, and one from United Cerebral Palsy—Central Arizona (UCP) in Phoenix.

Eligibility criteria for the original study included an age between 12 and 36 months, a diagnosis of cerebral palsy confirmed by a pediatric neurologist or pediatric rehabilitation specialist, and classified as Gross Motor Function Classification System (GMFCS) level I, II, or III as assessed by a physical therapist. Exclusion criteria included an MRI imaging study suggesting neuronal migration defects, co-morbidities that prevented the intensity or type of therapies or the evaluation, and any anticipated pharmacological intervention or procedures that might interfere with the study’s validity [5].

This current study included both retrospective and prospective components. The retrospective component used the final evaluation of the original study as the baseline. The prospective component evaluation was not designed to measure the effects of the original intervention.

### 2.2. Procedures in Two Phases

Phase 1 enrolled children who met the following criteria: (a) completed the 48-week protocol at least 1 year before recruitment for this follow-up study, (b) lived in either the Tucson or Phoenix metropolitan area, and (c) parental consent to an additional GMFM-66 and a PEDI-FS evaluation. The follow-up study began 10 months after the final child was enrolled in the original study. Thirty-nine eligible families were contacted by email; 18 (46%) agreed to participate. All but one child completed both the GMFM-66 and PEDI-FS assessments. One child completed only the PEDI-FS due to COVID-19 concerns. Unspent funds from the original study allowed the GMFM-66 to be included in Phase 1.

GMFM-66 [7] and PEDI-FS [8] procedures were identical to those used in the original study [5]. The treating therapist administered the GMFM-66, recording the assessment from two camera angles. Videos were encrypted and sent to a blinded physical therapist in Canada for scoring. The primary caregiver completed the PEDI-FS during the same session.

Phase 2 was initiated one year and 10 months after the last child was enrolled in the original study and involved children who had completed the 48-week protocol at the TMC facility at least one year after their last evaluation for the original study. The evaluation included the PEDI-FS and an open-ended questionnaire designed to obtain written statements from the primary caregiver. It included their perceptions of how their child was functioning and how the original study affected their child. Parents returned the completed materials by mail. Fourteen of the 21 eligible families (67%) participated. Funds were not available from the original study to conduct the GMFM-66.

The Institutional Review Boards providing oversight over each institution (PCH and TMC) responsible for this research approved the follow-up study.

### 2.3. Quantitative Measures

The GMFM-66 is a validated, criterion-referenced measure designed to assess gross motor function in children with CP aged 5 months to 16 years. A physical therapist scores each item on a 4-point scale: scores for each item are based on how well the child performs the skill: 0 (does not initiate), 1 (initiates < 10%), 2 (partially completes 10–99%), 3 (completes 100%), or NT (“not tested”) [9]. Total scores for the current report were calculated from the GMFM-66 Item Set scoring method, using the GMAE-2 software.

The PEDI-FS evaluates functional skills based on caregiver report. It is appropriate for children aged 6 months to 7 years with disabilities, including but not limited to CP. Items are scored dichotomously (1 = able, 0 = unable), without regard to efficiency or quality of performance. Functional skills are grouped into three domains: self-care, mobility, and social function. The PEDI-FS subset is used to assess each of the three domains as seen through the eyes of the primary caregiver through day-by-day observations in the child’s environment. The raw scores for each domain were calculated and translated into scaled scores (not adjusted for age) [9].

Although the GMFM-66 and PEDI-FS measure different constructs, prior research has demonstrated strong correlations between the GMFM-66 and the PEDI-FS mobility domain [10,11,12].

### 2.4. Qualitative Data Collection

Follow-up qualitative data were collected from 14 parents of 21 children treated at the TMC Pediatric Physical Therapy facility. When the parents were recruited, in addition to the PEDI-FS, they were also invited to respond to several open-ended survey questions. The questions addressed areas and skills where the child was doing well, areas and skills that the child was struggling with, the child’s functioning compared to expectations of the parents when their child was first diagnosed with CP, and the perceived influence the study had on their child’s functioning.

### 2.5. Qualitative Data Analysis

The qualitative analysis was conducted by a member of the research team with expertise in qualitative research methods and analysis. The first step consisted of the qualitative researcher creating a codebook of deductive and inductive codes. Deductive codes were based on interview questions, relevant literature, and conceptual frameworks for qualitative data analysis [13]. Additional inductive codes were created based on emergent themes from reviewing responses to the open-ended questions. The total group of codes covered a range of topics, including parents’ perceptions of their children’s physical, mental, cognitive, social, and academic functioning, parents’ expectations for their children’s functioning when they were first told that their child had CP, and parents’ experiences with the study intervention.

For the second step, the qualitative researcher created separate documents for the responses from each parent (i.e., one document per parent). The researcher uploaded the documents and entered the codebook in the qualitative software program ATLAS.ti 8 [14]. The researcher enhanced the distinctions between certain codes during this process as needed.

For the third step, the qualitative researcher created code reports for codes that were selected for analysis for the current study. The selected codes focused on parents’ perceptions of the impacts of the study intervention on their children’s functioning, barriers to treatment, and expectations of their children’s functioning when they were first diagnosed with CP. The researcher analyzed the code reports and identified emergent themes, patterns, and illustrative quotes. The qualitative researcher met with the lead investigators, reviewed the qualitative results, and determined if the responses were interpreted accurately.

## 3. Results

### 3.1. Sample Characteristics

Table 1 presents the demographic characteristics of the 23 children in this convenience sample. There were 12 female and 11 male participants. At the first therapeutic session of the original study, the mean age was 20.3 months (range 12 to 38 months). At the follow-up evaluations, the mean age was 50 months (range 37 to 91 months).

### 3.2. Change Scores Between 48 Weeks and Follow-Up

Seventeen (74%) of the 23 children who completed both the GMFM-66 and the PEDI-FS were in the first phase of the study. One child in the first phase only completed the PEDI-FS. The other 5 children who completed only the PEDI-FS were in the second phase of the study when funds were not available to administer the GMFM-66. Fourteen (61%) of the 23 children completed the PEDI-FS and an open-ended questionnaire. All three levels of severity were involved: GMFCS I (4 completing both tests and 2 completing only the PEDI-FS), GMFCS II (5 completing both tests and 2 completing only the PEDI-FS), and GMFCS III (8 completing both tests and 2 completing only the PEDI-FS). Table 2 presents the assessment scores at 48 weeks and follow-up for each participant by severity level. The follow-up periods varied for each child. The children’s ages at 48 weeks and follow-up are provided to document the time interval between the two assessment periods.

Because of the small sample size, only an overview and comparisons with developmental curves are credible. Based on the GMFM-66 percentile curves published by Hanna et al. [15], of the children with Level I GMFCS severity, 3 of 4 lost percentiles, but their GMFM-66 scores remained stable. The other child gained in scoring and remained on the same percentile. Of the children with Level II severity, all five gained percentiles. Of the eight children with Level III severity, 3 gained, 3 lost, and 2 remained on the same percentile curve. The primary caregivers’ perception of their child’s mobility as assessed by the PEDI-FS was compared with the curves published by Smits et al. [16]. The PEDI-FS Mobility subset at follow-up indicated that children with severity Levels II and III did not change their position relative to the mean, and those in Level I had lower positions. For the PEDI-FS Self-Care subset, more children in all three severity levels were below the mean at follow-up than at the 48-week evaluation. (Table 3).

### 3.3. Parents’ Perceptions of Study Impacts

Fourteen parents of the children from the TMC sample completed a survey with open-ended questions and reported their perceptions of how the study impacted their children’s functioning. Some parents reported changes in their children’s physical, mental, and social functioning from participating in the intervention study. The largest number of responses concerned improvements in different types of physical functioning. Some parents reported improvements in their children’s mobility, including walking and climbing stairs. A parent of a child classified as GMFCS Level III stated, “*She is able to walk. It also showed us how important constant therapy is to make an impact.*” Parents also reported improvements in their children’s dexterity, especially hand motor coordination. A parent of a child with GMFCS Level II described, “*He learned to use his paralyzed hand much more than he could*”. Some parents observed that their children displayed increased focus and motivation because of participating in the intervention. A parent of a child classified as GMFCS Level II said, “*I think it helped her slow down a little and focus on her movement.*”

A few parents attributed changes in their children’s mental and social functioning to participating in the intervention. Two parents described that their children developed goal-setting skills. A parent of a child classified as GMFCS Level I stated, “*It (intervention) taught her at a young age to work, adjust, practice, modify things to achieve a goal.*” A parent of a child classified as GMFCS Level III reported that the intervention provided opportunities for their child to engage in peer relationships. “*He was also afforded the opportunity of socialization with other peers his age (and therapists, of course) which was not an opportunity we were able to find elsewhere.*”

### 3.4. Parents’ Perceptions of Barriers to Treatment

Several parents reported that their child received different treatment by participating in the study compared to what they would have received in usual settings. The differences included the amount of treatment. The parents reported that the study intervention consisted of more sessions than in the usual treatment. A parent of a child classified as GMFCS Level III stated, “*The 5x/week was monumental in helping him get the needed repetition to practice whatever it was he was working on at the time. I believe it helped to build muscle memory more so than a single appointment once a week would do.*” Parents also described differences in the type of treatment that the study offered. A parent of a child classified as GMFCS Level III reported, “*Probably not the same amount (5x/week), and probably not the same type, but we definitely would have had therapies.*”

Many parents described that the study helped them overcome financial barriers to receiving more frequent treatment for their child. Several parents reported that health insurance plans did not pay for more frequent treatment. A parent of a child classified as GMFCS Level II stated, “*Therapy is expensive, and insurance covers a very small amount of therapy sessions.*” Some parents indicated that they did not have personal financial resources to pay for more frequent treatment. A parent of a child classified as GMFCS Level II reported, “*Financially, we could not have afforded the amount of therapy he received, and our insurance did not cover that amount of therapy.*”

### 3.5. Parents’ Expectations of Children’s Functioning When Diagnosed

Most parents reported that their children’s functioning was better than they expected when their children were diagnosed with CP. Parents shared that their negative expectations were influenced by information they received from healthcare providers. Parents reported that their child exceeded their expectations in several areas, including being independent. A parent of a child classified as GMFCS Level II said, “*Now that he is 8 years old, he is quite independent. He dresses, feeds himself, and makes his bed, brushes his teeth, and showers himself. All of the ADLs that we weren’t sure he was going to be able to do himself, he does*”. Several parents reported that their child exceeded their expectations regarding physical functioning and mobility. A parent of a child classified as GMFCS Level II stated, “*The doctors weren’t sure if he would walk. Now he runs fast. He can do so many things I wasn’t sure he would do.*” Another parent of a child classified as GMFCS Level III said, “*He is doing much better than I imagined based on how bad I was told the damage to his brain was*.” A few parents reported that their children exceeded their expectations related to verbal abilities and academic functioning. A parent of a GMFCS Level I child stated, “*I was afraid she would not be able to talk, write, spell, or read in school with her peers. This year in kindergarten, she is doing all of it!*” Other parents also spoke about how their children exceeded expectations regarding their ability to interact with peers. A parent of a child classified as GMFCS Level III reported, “*When first diagnosed, I believed he would never walk or do some of the things he can—I had such low hope of his being able to play with his peers*.”

There were two other groups of parents regarding expectations of their children. A couple of parents indicated that their children’s functioning was like what they expected when their child was first diagnosed with CP. Some parents were uncertain what to expect because healthcare providers were vague about functionality as the child developed. A parent of a child classified as GMFCS Level III stated, “*The only concrete fact we were told was from his MRI, that the region of his brain for cognitive function was unaffected. Our neurologist intentionally left it open, and in hindsight, it makes complete sense why, as every individual with cerebral palsy has different abilities.*”

## 4. Discussion

This is an observational study of a long-term follow-up of a convenience sample of 23 children who participated in a 48-week parallel-group randomized clinical trial that examined the effectiveness of an intense intervention of the Perception-Action Approach [5]. There was a considerable difference in the change scores in the children for both evaluation assessment tools: the GMFM-66 and the PEDI-FS. The general trend for the GMFM-66 was an improvement in overall function, with the most consistent and greatest improvements in children classified as severity level GMFCS II.

The change scores of the current cohort are interpreted using sets of curves documented in the literature. In 2002, Rosenbaum et al. published longitudinal prognostic curves for gross motor function in children from 1 to 13 years based on age and change scores in the GMFM. They followed 657 children serially for up to 4 years and constructed separate curves based on age and GMFM scores for each of the five severity levels [15]. In 2009, Hanna et al. developed percentile curves from Rosenbaum’s data, and our follow-up data were compared with those curves [16]. As expected, the curves were different for the different GMFCS levels. Eight of the 17 children who had GMFM-66 evaluations gained percentiles. All children classified as GMFCS Level II severity gained percentiles. In 2019, Smits et al. published curves of longitudinal data on the PEDI-FS of 551 children with confirmed CP. Based on 1754 PEDI-FS assessments, they published developmental curves according to age and scores on the Mobility and Self-Care sub-sets for each of the five severity levels [17]. It is not surprising that comparing our data with those developmental curves shows the variable expressivity of children with CP and variable differences in their responses to physical therapeutic interventions.

The current qualitative findings provided additional insights into the benefits of an intense P-AA intervention. Many parents perceived improvements in their children’s functioning, including physical, mental, and social functioning, because of participating in the intense intervention study. Parents reported improvements in different types of physical functioning, including mobility and dexterity. A couple of parents described that their children developed goal-setting skills because of the intervention. One parent reported that the intervention provided opportunities for their child to engage in peer relationships. To date, there are no published studies on caregivers’ perceptions of the impacts of P-AA on their children’s functioning. In Novak et al.’s review of systematic reviews of the best available evidence of interventions for preventing and managing CP, while they indicated that families prioritize evidence-based therapeutic interventions, with similar approaches to P-AA (early, collaborative, goal-directed, family-centered approaches that build caregiver capacity), the review does not address parents’ perceptions of specific interventions [4].

Parents’ open-ended responses on the survey also provided insights into barriers to treatment for children with CP and other challenges that families face. Several parents reported that the study intervention consisted of a greater number of sessions (5 times a week) compared to the usual treatment. The study intervention helped parents overcome financial barriers to accessing more frequent treatments for their child, as health insurance plans did not pay for an increased level of therapy. Some parents noted that they did not have the financial resources to pay for the intense treatment out of pocket. One of the major themes from a meta-synthesis of qualitative studies on caregivers’ experiences of having a child with CP was the need for healthcare information and financial aid [18]. Caregivers reported that money is needed to pay for expensive medications, therapies, and transportation to care facilities. Sometimes caregivers had to make compromises to care for their child’s healthcare and financial needs. Another review of qualitative research on experiences and the needs of parents of children with CP documented that parents frequently mentioned having a lack of financial support [19]. And if financial support was available, the amount was insufficient. The lack of support resulted in financial burdens for the family and prevented them from accessing necessary care for their children.

Most parents reported that their children’s functioning was better than they expected when their children were diagnosed with CP. Parents indicated that their negative expectations were influenced by information received from healthcare providers. Parents reported that their child exceeded their expectations in several areas, including independent daily living skills, physical functioning and mobility, verbal abilities, and academic functioning. A few parents reported that their children’s functioning matched their expectations when they were diagnosed with CP. Some parents were uncertain what to expect because healthcare providers were vague about functionality as the child developed. Past research has highlighted that parents with children with CP have described similar challenges with healthcare providers. A qualitative study on parents’ views on early diagnosis of CP showed that parents perceived that doctors are hesitant to give a diagnosis of CP because an accurate prognosis of the child’s future development is difficult to give [20]. Some parents indicated that an uncertain prognosis created stress and anxiety. A systematic review by Elangkovan and Shorey showed that some parents reported that the lack of information, transparency, and open communication from healthcare providers contributed to feeling helpless, created mistrust, and increased their fears and worries [19]. Another qualitative investigation found that parents with infants diagnosed at high risk for CP expressed a desire for honest support and normalization of their experiences from healthcare providers [21].

## 5. Strengths and Limitations

Strengths: The current study has several strengths. Except for surgical interventions like selective dorsal rhizotomy, [22] there are few follow-up studies of children with CP following a clinical intervention. In those that are published, the follow-up is much shorter than in this observational study. Another strength was that the cohort of the original investigation was younger than in most other studies on children with CP. To our knowledge, there are limited, if any, mixed-method follow-up studies on very young children with CP. Parents’ responses to the open-ended survey questions deepened our understanding of the wide range of improved functioning that children with mild-to-moderate spastic CP might experience following intensive physical and occupational therapeutic intervention. The parents’ responses also confirmed and provided additional insights into the challenges that parents with children with CP experience with healthcare providers and healthcare services.

Limitations: The current study is an observational report, not a formalized research study with a specific hypothesis to test causal relationships. A serious limitation that is present in most investigations on children with CP is the heterogeneity of this group of children. The sample of children was a small convenience sample that represented just over half of the children eligible for the follow-up. Data was not collected on any therapies the children may have received after completing the 48-week protocol of the original study, which may have influenced functionality at the long-term follow-up assessment. There were differences in the children’s ages at baseline and follow-up evaluations, and variable intervals between baseline and follow-up scores. There were no controls. But the changes were compared with published percentile curves based on age that depict changes in functional skills of children with CP. Qualitative data was only collected from the TMC sample. Parents from additional sites from the original investigation may have had different perceptions of their children’s functioning and their expectations. Only broad statements can be made relative to the quantitative and qualitative data.

## 6. Implications for Practice

The current study supports that more intense and early intervention for children with mild-to-moderate levels of spastic CP results in positive changes in functioning that are sustained long-term for some but not all children. There is a need to increase children’s access to such interventions by offering support services, such as financial aid and transportation. This study underscores the diversity in the functioning of children diagnosed with spastic CP and the generally unpredictable nature of the children’s developmental trajectory. Several parents reported that their children’s functioning exceeded their expectations and was influenced by interactions with healthcare providers in a variety of areas, including physical functioning, mobility, independence, verbal abilities, academic functioning, and social relationships.

Based on parents’ responses to the open-ended survey questions, we recommend improving physician and healthcare providers’ training to better handle the complexities of early diagnosis of CP. Physicians should avoid being too optimistic or too pessimistic when giving the diagnosis and prognosis of CP to parents, and when counseling on how to care for their children. Overly negative prognosis and expectations may jeopardize a child’s reaching their full potential. Parents may not seek or commit to resources and support that could benefit them, unintentionally avoiding resources and experiences that could enhance their child’s development. There is also a risk that a too positive outlook from healthcare providers may frustrate both the child and the parents and contribute to disappointment and other negative experiences. Research has found that parents want to receive honest, transparent, and open communication from healthcare providers [21]. From the other perspective, clinicians report that they need to gain skills in how best to advise parents in giving a prognosis in such a complex disorder [21]. Clinicians also report lacking training in mental health and difficulties in responding to parental grief and distress, and demand clear answers when faced with uncertainty about their children’s future. The qualitative findings from our study and others have identified these gaps in the training of healthcare providers who are involved in the assessment and treatment of children with CP and should be addressed. As with any child, each milestone a child with CP achieves should be acknowledged and the next one strongly encouraged.

## 7. Recommendations for Future Research

Future follow-up studies on intense, early interventions for children with spastic CP should implement more rigorous quantitative/qualitative methods. The length of follow-up should be standardized for all study participants. There should be consistency in data collection methods with all study participants. There is also a need for more qualitative research on children’s functioning after receiving intensive, early interventions. There is a large body of qualitative research on the experiences and needs of parents with children with CP, but relatively few qualitative and mixed-methods studies examining the impact of specific types of CP interventions. There is also a need for more investigations on the complex experiences that parents have with healthcare providers and the diagnosis, prognosis, and early interventions for children with CP. Morgan et al. recommended future research with more diverse samples of parents to more fully understand parents’ experiences with early intervention [20]. It was noteworthy that most of the parents in the current study reported that their children’s functioning in numerous areas exceeded the expectations given by healthcare providers. An important area of study is whether parents’ expectations are associated with different healthcare-seeking behaviors, services, and children’s outcomes. Given the complexity of caring for a child with CP, future CP researchers should consider embracing principles and methods that promote greater engagement of parents in the research process. Headrick and colleagues conducted a study on consumer involvement in research and found that parents believed that parent partnership in research would benefit both the researchers and the consumers involved in the studies [23].

## 8. Conclusions

This study identified several areas in cerebral palsy (CP) research and healthcare services that need to be improved to address the needs of children and their caregivers. Although prognostic curves are helpful, the long-term functional outcome of children diagnosed with cerebral palsy is unfortunately problematic. Each child is unique, and each follows their own trajectory. Based on prognostic GMFM-66 developmental curves by age for children with CP, seven of the 17 children who had GMFM evaluations showed greater than expected improvement (all 5 with GMFCS II), four met the expected improvement, and six did not. More research is needed on the long-term effects of intensive treatment interventions for children with spastic CP. Healthcare providers need to engage in more honest and balanced communication and encourage children and their families to aim for realistic and feasible goals. Many children in the present study exceeded their care providers’ and their guardian/parents’ expectations, providing cautious credibility for more positive prognoses by healthcare providers.

## Figures and Tables

**Table 1:** Demographics and sample size post-stratified by severity level for follow-up GMFM-66 and PEDI-FS participants.

*Demographic Characteristics*	*Sample Size*
Total Convenience Sample (N)	23 children
Mean Age (months)	20.3
Gender	52% Female (n = 12); 48% Male (n = 11)
*GMFCS Level & Evaluation Completed*	
GMFCS I GMFM-66 (n)	4
GMFCS I PEDI-FS (n)	6
GMFCS II GMFM-66 (n)	5
GMFCS II PEDI-FS (n)	7
GMFCS III GMFM-66 (n)	8
GMFCS III PEDI-FS (n)	10

**Table 2:** Phase 1 individual children (n = 17) post-stratified by severity levels, GMFCS I, II, and III. Baseline age in months, actual GMFM-66 score, and percentile (Hanna et al.) at the end of a PT and OT intervention that included an intense phase; Follow-up age in months, actual GMFM-66 score, and percentile; Change in percentile (↑ = increased, ↓ = decreased, and - = no change).

GMFM-66 GMFCS I			GMFM-66 GMFCS II			GMFM-66 GMFCS III		
Baseline	Follow-Up	Change	Baseline	Follow-Up	Change	Baseline	Follow-Up	Change
46 mos.	78 mos.		28 mos.	86 mos.		36 mos.	91 mos.	
75.3	72.2	**↓**	60.9	80	**↑**	40.4	42.8	**↓**
75%	10%		95%	97%		10%	5%	
36 mos.	62 mos.		27 mos.	77 mos.		29 mos.	71 mos.	
80	80.9	**↓**	55.6	74.7	**↑**	50.9	54.9	**↓**
97%	60%		75%	97%		95%	60%	
25 mos.	52 mos.		27 mos.	83 mos.		32 mos.	56 mos.	
69.9	72.2	**↓**	61.8	80	**↑**	59.1	79.1	**-**
90%	50%		95%	97%		>97%	>97%	
29 mos.	50 mos.		24 mos.	69 mos.		26 mos.	49 mos.	
69.2	81.9	**-**	66	82.9	**↑**	47.9	64	**↑**
90%	90%		97%	>97%		60%	>97%	
			34 mos.	65 mos.		32 mos.	49 mos.	
			45.3	53.1	**↑**	28	39.2	**↑**
			10%	25%		<3%	3%	
						22 mos.	64 mos.	
						56.6	69.2	**-**
						>97%	>97%	
						37 mos.	64 mos.	
						50.6	50.9	**↓**
						95%	50%	
						27 mos.	37 mos.	
						33.9	35.3	**-**
						<3%	<3%	

**Table 3:** Comparing our baseline and follow-up data with the developmental curves for the PEDI-FS Mobility and Self-care domains from Smits et al. [17].

**Smits PEDI-FS Mobility [17]**	**Below the Mean**		**At or Near the Mean**		**Above the Mean**	
	**Baseline**	**Follow-up**	**Baseline**	**Follow-up**	**Baseline**	**Follow-up**
GMFCS I		3/6	1/6	1/6	5/6	1/6
GMFCS II		3/7	1/7	1/7	6/7	3/7
GMFCS III	4/10	4/10	1/10	1/10	5/10	5/10
**Smits PEDI-FS Self-Care [17]**	**Below the Mean**		**At or Near the Mean**		**Above the Mean**	
	**Baseline**	**Follow-up**	**Baseline**	**Follow-up**	**Baseline**	**Follow-up**
GMFCS I		4/6	1/6		5/6	2/6
GMFCS II	1/7	3/7	1/7	4/7	5/7	
GMFCS III	3/10	6/10	3/10		4/10	4/10

## Data Availability

The original contributions presented in this study are included in the article. Further inquiries can be directed to the corresponding author.
